# Better Operating Room Ventilation as Determined by a Novel Ventilation Index is Associated With Lower Rates of Surgical Site Infections

**DOI:** 10.1097/SLA.0000000000005670

**Published:** 2022-08-10

**Authors:** Bernard Surial, Andrew Atkinson, Rüdiger Külpmann, Arnold Brunner, Kurt Hildebrand, Benoît Sicre, Nicolas Troillet, Andreas Widmer, Eveline Rolli, Judith Maag, Jonas Marschall

**Affiliations:** * Department of Infectious Diseases, Inselspital, Bern University Hospital, University of Bern, Bern, Switzerland; † Lucerne University of Applied Sciences and Arts, Engineering and Architecture, Horw, Switzerland; ‡ Brunner Consulting, Pfaeffikon ZH, Switzerland; § LET GmbH, Wettswil am Albis, Switzerland; ∥ Service of Infectious Diseases, Central Institute, Valais Hospitals, Sion, Switzerland; # Department of Infectious Diseases, University Hospital Basel, Basel, Switzerland; ¶ Swissnoso, National Center for Infection Control, Bern, Switzerland; ** Division of Infectious Diseases, Washington University School of Medicine, St. Louis, MO

**Keywords:** laminar air flow, operating room ventilation, prevention, surgical site infections, ventilation index

## Abstract

The aim was to assess the impact of operating room (OR) ventilation quality on surgical site infections (SSIs) using a novel ventilation index.

**Background:**

Previous studies compared laminar air flow with conventional ventilation, thereby ignoring many parameters that influence air flow properties.

**Methods:**

In this cohort study, we surveyed hospitals participating in the Swiss SSI surveillance and calculated a ventilation index for their ORs, with higher values reflecting less turbulent air displacement. For procedures captured between January 2017 and December 2019, we studied the association between ventilation index and SSI rates using linear regression (hospital-level analysis) and with the individual SSI risk using generalized linear mixed-effects models (patient-level analysis).

**Results:**

We included 47 hospitals (182 ORs). Among the 163,740 included procedures, 6791 SSIs were identified. In hospital-level analyses, a 5-unit increase in the ventilation index was associated with lower SSI rates for knee and hip arthroplasty (−0.41 infections per 100 procedures, 95% confidence interval: −0.69 to −0.13), cardiac (−0.89, −1.91 to 0.12), and spine surgeries (−1.15, −2.56 to 0.26). Similarly, patient-level analyses showed a lower SSI risk with each 5-unit increase in ventilation index (adjusted odds ratio 0.71, confidence interval: 0.58–0.87 for knee and hip; 0.72, 0.49–1.06 for spine; 0.82, 0.69–0.98 for cardiac surgery). Higher index values were mainly associated with a lower risk for superficial and deep incisional SSIs.

**Conclusions:**

Better ventilation properties, assessed with our ventilation index, are associated with lower rates of superficial and deep incisional SSIs in orthopedic and cardiac procedures. OR ventilation quality appeared to be less relevant for other surgery types.

Surgical site infection (SSI) is the most common preventable complication among patients who undergo surgery and is associated with substantial morbidity and healthcare cost.^1^–^4^ Prevention of SSIs requires a multimodal bundle approach: While efforts such as antimicrobial prophylaxis and surgical hand hygiene have been shown to be effective measures to prevent SSIs,^5^–^7^ and multiple other measures are being advocated in prevention guidelines, the role of operating room (OR) ventilation during the index surgery proved to be controversial.^8^–^10^

Laminar air flow systems are designed to move filtered air uniformly with little or no turbulence into the operating field to minimize microbial wound contamination through air.^11^ Although studies showed that laminar air flow systems reduce the bacterial load in an operating field,^12^ these findings did not translate into decreasing SSI rates in most clinical studies, as summarized in a large meta-analysis of observational studies.^13^ However, all included studies relied on an oversimplified distinction between laminar air flow and conventional ventilation systems. These studies provide insufficient data on technical characteristics such as ceiling panel size, air flow, and presence or absence of objects above the operation field, which play an important role in the flow dynamics and hence in the capacity of ventilation systems to reduce microbial contamination.^14^–^16^

To fill this research gap, we aimed to characterize the ventilation quality of Swiss ORs using a novel ventilation index encompassing a range of ventilation characteristics, and assessed the impact of ventilation quality on SSI rates using data from the national SSI surveillance database.

## METHODS

### Study Setting and Participants

This cohort study is based on the Swiss national SSI surveillance program (www.swissnoso.ch) including 168 of the 276 hospitals in Switzerland,^17^ in which data on at least 3 procedure types per participating hospital are routinely recorded for all patients who undergo surgery. In addition to in-hospital surveillance, postdischarge surveillance is performed at 30 days after all procedures, and again at 1 year for individuals undergoing implant surgery, with complete 1-year follow-up data available for more than 90% of operations. Trained infection control nurses perform regular systematic reviews of patient charts and standardized postdischarge phone interviews, supervised by infectious disease specialists. Here, we included all adult patients with complete follow-up between January 2017 and December 2019. Due to the high proportion of children undergoing appendectomy, we excluded this procedure from our analyses.

For the study, all hospitals participating in the SSI surveillance program were contacted and asked to provide detailed technical information for all ORs in which procedures captured by the national surveillance were performed, including 2 standardized photographs to confirm the plausibility of the data entry (Fig. S1, Supplemental Digital Content 1, http://links.lww.com/SLA/E164). All hospitals that provided measurements on at least 1 OR were included in this analysis.

The Cantonal Ethics Committee of Bern, Switzerland (Project ID 2019-00294) approved the study. Patients were informed about their inclusion in the SSI surveillance on admission and given the opportunity to opt out. This study follows the Strengthening the Reporting of Observational Studies in Epidemiology (STROBE) reporting guideline.^18^

### Ventilation Index

Previous laboratory experiments by members of our team examined the influence of specific ventilation system characteristics on air quality in ORs. Those experiments were performed in a real-life replication of an OR (7.2×6.5×3.2 m), in which specific changes both to the ventilation system and the operating field could be applied and tested. Under varying controlled simulations, optical particle measurements were obtained from within the operating field (in accordance with the Swiss and German industry recommendations), and a ratio was determined by comparing the measurements to a reference value. The resulting level of protection describes the ventilation induced air displacement and particle dilution, which correlate directly with the reduction in microbial burden.^19^ On the basis of those results, we created a ventilation index that is influenced by the size of the supply air unit, the delivered air flow into the room, and affected negatively for factors that potentially cause turbulence (such as design and position of OR lamps, location of return air outlets, and table position). The index was calculated differently for ORs with and without laminar air flow units, with higher values reflecting less turbulent air displacement properties (Table 1). While SSI rates were available on the procedure type and hospital level, ventilation index values were collected on an OR level. For each hospital, we calculated overall and procedure-specific ventilation indexes using the mean values of the respective ORs.

**TABLE 1:** Calculation of the Ventilation Index

		Points assigned	
Item	Quality	Laminar air flow unit	Conventional unit
Air flow (m^3^/h)		1 pt per 1000 m^3^/h	1 pt per 1000 m^3^/h
Size of ceiling unit	Area ≥6 m^2^	4	0
	Area <6 m^2^	2	0
Location of air return outlets	Symmetrical, floor	0	0
	Symmetrical, close to ceiling	−2	−1
	Asymmetrical	−4	−2
Air guide at ceiling unit	Long guide	0.5	0
	Short guide	0	0
	No guide	−1	0
Operating room lamps	Stand-alone lamps	0	0
	Lamp allowing air passage	−2	0
	Impermeable lamp	−4	0
Patient-table position	Movable	0	0
	Stationary	−1	0

pt indicates point.

### Outcomes

The primary outcome was the hospital-specific rate of any SSI (including superficial incisional, deep incisional, or organ/space infection) according to National Healthcare Safety Network definitions from the Centers for Disease Control and Prevention.^20^ All SSIs at the 30-day or 1-year time points were included, and SSI rates were standardized using the National Nosocomial Infections Surveillance (NNIS) risk index, which accounts for differences in the American Society of Anesthesiology (ASA) score, the wound contamination class (clean/clean-contaminated or contaminated/infected), and whether the procedure duration was above the 75th percentile.^21^ Standardized SSI rates were calculated for each hospital, and individually for each type of surgical procedure within a given hospital. In addition to hospital-specific SSI rates, we also calculated the individual patient-level risk for any SSI in a secondary analysis.

### Statistical Analyses

To explore the association between the ventilation index and hospital-specific SSI rates, we fitted hospital-level multivariable linear regression models, adjusted for procedure type (cesarean section, cardiac, colorectal, hernia, hysterectomy, knee and hip, spine, and upper gastrointestinal surgery), including weights for the number of procedures performed during the observation period. As each hospital could contribute data for more than 1 procedure type, we calculated sandwich-type “robust” standard errors to account for intrahospital correlation. In addition, separate weighted univariable linear regression models were performed individually for each procedure type. Adjusted odds ratios (aOR) were calculated using patient-level data by fitting multivariable generalized linear mixed effect models with a logit link function. The procedure-specific mean ventilation index for each hospital was used as exposure in these models, fitted with 3 distinct adjustment sets: the first model included the procedure type and all components from the NNIS risk index; the second model added whether the procedure was elective versus urgent, use of adequate antibiotic prophylaxis (within 120 minutes prior to the incision for fluoroquinolones and vancomycin, and within 60 minutes for other antibiotics), and the patient's age and sex; and the third model additionally comprised hospital-level information such as hospital size (by number of beds) and setting (public, private, or university hospital). To account for correlation within hospitals, all patient-level models included a random intercept for each hospital. We repeated the overall patient-level analysis for each type of infection (superficial, deep incisional, and organ/space) as outcome. As the proportion of missing data was low (below 0.5% for all variables included), analyses were performed on complete cases. Statistical analyses were performed using R, version 4.1.1 (R Foundation for Statistical Computing, Vienna, Austria).^22^

### Sensitivity Analyses

We explored the robustness of our results using several sensitivity analyses on the hospital level. As our data did not allow matching patients and ORs directly, we relied on aggregating ventilation indexes within hospitals. To explore the impact of the aggregation method, we repeated the hospital-level analyses using the minimum and maximum ventilation index of all ORs, respectively. In addition, we performed an analysis including only hospitals with homogeneous ventilation indexes (defined as having a standard deviation of ventilation indexes below the 75th percentile) to limit the influence of ORs with very good or very poor ventilation properties. We also performed a hospital-level analysis restricted to public hospitals to limit the potential bias of differences in case mix and resources.

## RESULTS

### Description of Participating Hospitals

Out of 168 hospitals contacted, 51 hospitals from all parts of Switzerland completed our survey, and 48 (94%) provided enough details for us to calculate the ventilation index and had SSI data available. Another hospital was excluded since only data on appendectomies were available. Most of the 47 included hospitals were public (26, 55.3%), followed by private (17, 36.2%) and university hospitals (4, 8.5%). Thirty-one hospitals (66.0%) had less than 200 beds, 10 hospitals had 200 to 499 beds (21.3%), and 6 hospitals (12.8%) had 500 or more beds. Compared with hospitals participating in the SSI surveillance program that were excluded from our analyses (n=121), those included were more likely to be university hospitals (8.5% vs. 1.7%), and less likely to be private (36.2% vs. 40.2%). The number of beds did not differ substantially between included and excluded hospitals, although the proportion of larger hospitals (500 beds and more) was higher in the included set of hospitals (12.5% vs. 5.1%).

**FIGURE 1:**
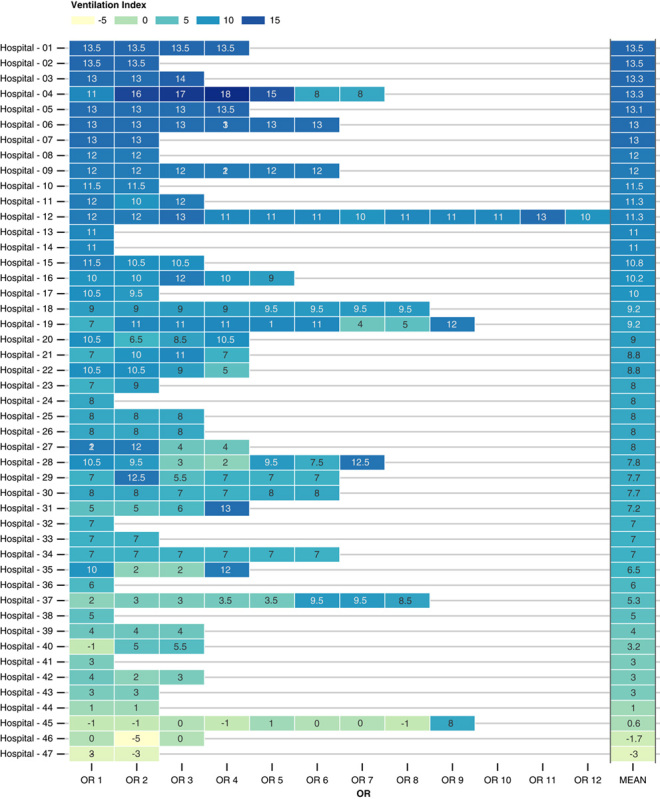
Ventilation indexes of all 182 operating rooms from 47 participating hospitals. Each square indicates a distinct operating room (OR) within a hospital. The ventilation index summarizes properties of laminar air flow quality, with higher ventilations indexes implying less turbulent air displacement. For each hospital, ventilation quality was summarized by calculating the mean index of all ORs.

Ventilation indexes were calculated for 182 ORs in the 47 included hospitals. The mean ventilation index was 8.3 [95% confidence interval (CI): 7.7–9.0] and ranged from −5 to 18. Figure 1 shows a detailed description of ventilation indexes for all ORs and the hospital-specific mean ventilation index. Ventilation indexes disaggregated by procedure types are presented in Figure S1, Supplemental Digital Content 1, http://links.lww.com/SLA/E164.

### Hospital-level Analysis

**FIGURE 2:**
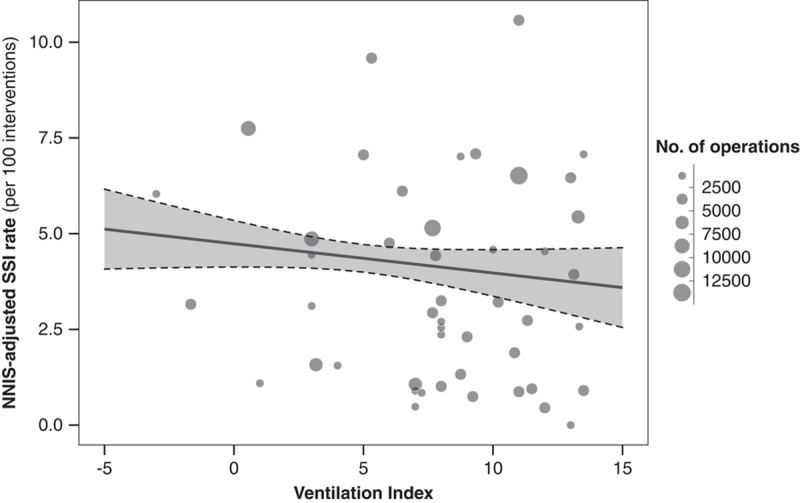
Association between ventilation index and National Nosocomial Infections Surveillance (NNIS)-adjusted surgical site infection rate on the hospital level. Each point represents one hospital, and its size correlates with the number of procedures performed within that hospital between 2017 and 2019. The line and 95% confidence interval ribbon show the association between ventilation index and the surgical site infection rates, adjusted for the number of procedures and the type of surgery performed. SSI indicates surgical site infection.

In hospital-level analyses, an increase in ventilation index was not associated with an overall decrease in NNIS-adjusted SSI rates (a change in SSI rate per 5 steps increase in ventilation index: −0.38 infections per 100 interventions, 95% CI: −1.04 to 0.28, Fig. 2). In procedure-stratified analyses, an increase of 5 units in ventilation index was associated with significant decreases in SSI rates for knee and hip arthroplasty (−0.41 infections per 100 interventions, 95% CI: −0.69 to −0.13). Similarly, increases in ventilation indexes were associated with lower SSI rates in cardiac (−0.89 infections per 100 interventions, 95% CI: −1.91 to 0.12) and in spine surgeries (−1.15, 95% CI: −2.56 to 0.26), albeit confidence intervals included a null effect for the latter 2 procedure types. No changes in SSI rates were observed in upper gastrointestinal and colorectal surgeries, cesarean sections, hysterectomies, and hernia repairs (Table 2). Exploring the role of implants, ventilation index was associated with decreases in infections among spine surgeries without the use of implants (−1.13 infections per 100 interventions, 95% CI: −2.16 to −0.11) and in those with implants (−1.01 infections per 100 interventions, 95% CI: −4.08 to 2.06) although the latter was not statistically significant.

**TABLE 2:** Hospital-Level Analysis of Ventilation Index and SSI Rate, by Surgery Type

Surgery Type	Change in SSI per 100 Interventions (95% CI)*	*P*
Cesarean section	−0.02 (−0.51 to 0.48)	0.94
Cardiac	−0.89 (−1.91 to 0.12)	0.072
Colorectal	−0.30 (−1.91 to 1.31)	0.71
Hernia repair	−0.02 (−0.35 to 0.32)	0.91
Hysterectomy	0.36 (−0.90 to 1.62)	0.51
Knee and hip arthroplasty	−0.41 (−0.69 to −0.13)	*0.005*
Spine (± implant material)	−1.15 (−2.56 to 0.26)	0.092
Upper GI	−0.24 (−0.99 to 0.51)	0.51

* Changes in SSI rates, per 5 units increase in the ventilation index.

CI indicates confidence interval; GI, gastrointestinal; SSI, surgical site infection.

### Patient-Level Analysis

Between January 2017 and December 2019, 163,740 procedures were included in our analyses. Patient and hospital characteristics are summarized in Table S1, Supplemental Digital Content 1, http://links.lww.com/SLA/E164. Within this 3-year period, 6971 SSIs (4.3%) were identified: 2399 superficial incisional infections (34.4%), 1109 deep incisional infections (15.9%), and 3172 organ/space infections (45.5%).

**FIGURE 3:**
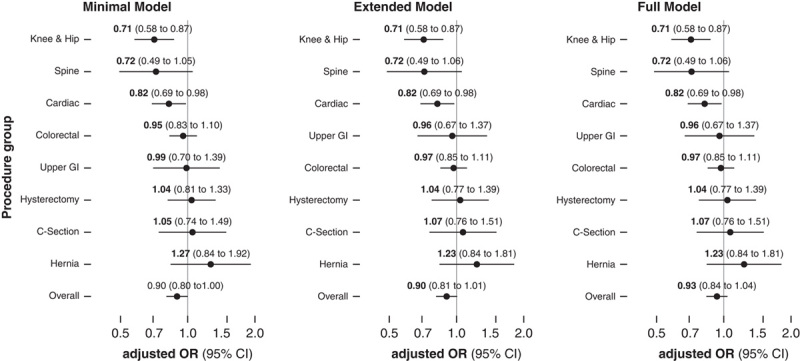
Patient-level analysis assessing the odds ratios of surgical site infections, overall and stratified by surgery type, per 5 units increase in ventilation index. The minimal model is adjusted for all components of the National Nosocomial Infections Surveillance (NNIS) risk index, and the intervention type. The extended model includes the same covariates as the minimal model, and additionally elective versus urgent surgery, adequate timing of antibiotic prophylaxis (within 120 minutes for fluoroquinolones and vancomycin, and within 60 minutes for other antibiotics), age, and sex. The full model includes all covariates, including hospital size and type (public, private, and university). CI indicates confidence interval; C-section, cesarean section; GI, gastrointestinal; OR, odds ratio.

**FIGURE 4:**
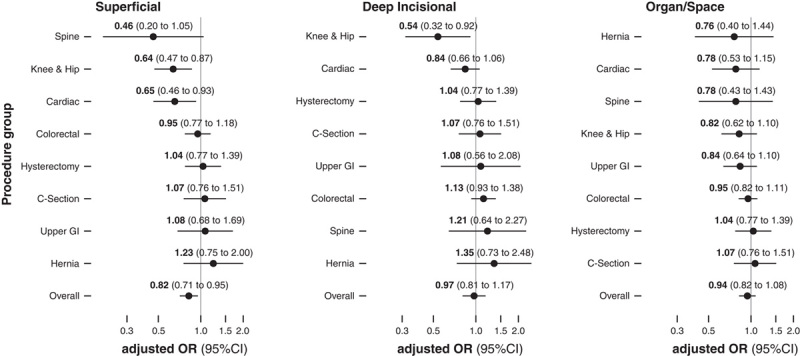
Patient-level analysis assessing the odds ratios of different types of surgical site infections, overall and stratified by surgery type, per 5 units increase in ventilation index. Patient-level analysis for each type of surgical site infection. The models are adjusted for all components of the National Nosocomial Infections Surveillance (NNIS) risk index, elective versus urgent surgery, adequate timing of antibiotic prophylaxis (within 120 minutes for fluoroquinolones and vancomycin, and within 60 minutes for other antibiotics), age, and sex. CI indicates confidence interval; C-section, cesarean section; GI, gastrointestinal; OR, odds ratio.

In models adjusting for variables from the NNIS risk index (minimal model), a 5-unit increase in ventilation index was associated with an overall lower risk for SSIs (aOR: 0.90, 95% CI: 0.80–1.00). In subgroup analyses, the largest reductions were again observed in knee and hip, spine, and cardiac surgery, with no clear associations observed in other surgery types. After additionally adjusting for whether the procedure was elective or not, the timing of antibiotic prophylaxis, patient age and sex (extended model), and after taking hospital size and type into account (full model), reductions in SSI rates associated with higher ventilation indexes remained significant for knee and hip, spine and cardiac surgery, but confidence intervals of overall estimates included a null effect (Fig. 3). In patient-level analyses adjusted for the intervention type, the components of the NNIS risk index, emergent indication, use of antibiotic prophylaxis, age, and sex, a 5-unit increase in the ventilation index was associated with overall lower rates of superficial (aOR for all procedures 0.82, 95% CI: 0.71–0.95), but not with deep incisional (aOR for all procedures 0.97, 95% CI: 0.81–1.17), or organ/space infections (aOR: 0.94, 0.82–1.08). Subgroup analyses showed lower rates of superficial incisional infections for knee and hip, cardiac and spine surgeries, and deep incisional infections for knee and hip surgeries with higher ventilation indexes. There were no significant associations with organ/space infections (Fig. 4).

### Sensitivity Analyses

Repeating the hospital-level analyses using minimum and maximum ventilation indexes instead of the mean ventilation index per hospital did not alter our findings substantially (Table S2, Supplemental Digital Content 1, http://links.lww.com/SLA/E164). In addition, results remained similar when only hospitals with homogeneous ventilation indexes were considered (knee and hip: −0.39 infections per 100 interventions, 95% CI: −0.71 to −0.06; spine: −1.15 per 100 interventions, 95% CI: −2.56 to 0.26; cardiac: −1.53, 95% CI: −4.8 to 1.7). As ventilation indexes were higher in private [median: 10.8, interquartile range (IQR): 7.3–11.5] and university hospitals (11.0, IQR: 8.0–11.0) compared with public hospitals (7.7, IQR: 3.0–9.2, *P*<0.001), we repeated the hospital-level analyses restricted to public hospitals. Similar to the full hospital-level analysis, ventilation indexes were not associated with an overall change in SSI rate (−0.20 infections per 100 interventions, 95% CI: −0.75 to 0.36), however, the association remained statistically significant for knee and hip surgeries (−0.47 infections per 100 interventions, 95% CI: −0.80 to −0.14).

## DISCUSSION

In this nationwide study, OR ventilation properties assessed using a novel ventilation index varied markedly between participating hospitals and between individual ORs within these hospitals. We observed lower rates of SSIs when orthopedic and cardiac procedures were performed in hospitals with better OR ventilation properties and higher ventilation indexes. This association was observed in hospital-level and patient-level analyses and was mainly driven by higher rates of superficial and deep incisional infections associated with lower ventilation indexes. Importantly, the strongest associations were present for knee and hip arthroplasty, spine, and cardiac surgeries, whereas no clear signal was present for gynecologic or abdominal surgeries, indicating that laminar air flow might be less important in these procedure types.

The finding that ventilation quality is associated with lower SSI rates is supported by microbiological studies which demonstrated substantial reductions in bacterial counts within the operating field when laminar air flow was used.^12^,^23^,^24^ However, clinical studies evaluating the role of laminar air flow in orthopedic surgery did not show a beneficial impact on SSI rates.^13^,^25^–^27^ Since no standardized metric of OR ventilation quality was available to date, previous studies mainly classified ventilation types into laminar air flow versus conventional ventilation. However, this distinction does not capture the complexity of OR ventilation, given that properties such as ceiling panel size^14^ and air flow play a crucial role in generating a truly laminar air flow. For instance, lower SSI rates were observed with high-volume but not with low-volume laminar air flow ventilation in an observational study performed in Norway, illustrating the importance of incorporating all components of OR ventilation when determining the impact of ventilation on SSI rates.^28^ To overcome this problem, we developed a novel and easy-to-calculate ventilation index, which encompasses a range of ventilation system characteristics. From an infection prevention perspective, such a tool may help assess and compare OR ventilation systems and provide a basis for assigning selected procedures to ORs with better ventilation characteristics.

While several previous studies explored the role of ventilation on the occurrence of deep incisional or organ/space infections,^13^,^25^,^26^,^29^,^30^ very little is known about the impact of ventilation quality on superficial incisional infections. One older study from Turkey observed a higher rate of sternal wound infections when operations were performed in rooms with out-of-date ventilation technology.^31^ Confirming and extending these findings, our study indicates that the lower rate of SSIs associated with improved laminar air flow properties was mainly driven by changes in the rate of superficial incisional infections followed by deep incisional, but not by organ/space nor infection of the implant itself.

SSI rates for procedures other than orthopedic and cardiac surgeries did not differ in function of the ventilation index in our study, indicating that ventilation may play a lesser role in these procedure types. These findings suggest that priorities of improving ventilation could be given to ORs where orthopedic and cardiac surgeries are performed, while maintaining conventional ventilation in other ORs to limit costs. However, whether laminar air flow ventilation actually leads to increases in operational costs remains a matter of debate.^32^–^34^

The present study included a large number of operations from a nationwide, well-characterized surveillance cohort of SSIs. Ascertainment of infections by trained infectious disease physicians with outcome assessment extending to 1 year after the intervention further strengthens the validity of our results. In addition, accounting for antibiotic prophylaxis in our analyses avoids one of the major limitations of previous studies which did not include this information.^25^,^26^ Finally, findings were robust across a wide range of sensitivity analyses, including an analysis restricted to public hospitals, thereby reducing the potential bias due to differences in resources and case mix between hospitals.

Some limitations should be noted. The surveillance database did not record the individual OR in which the procedure was performed. Therefore, we had to rely on aggregated ventilation indexes per hospital as the exposure, which could have led to exposure misclassification. However, the results remained robust when using different aggregation methods, including when limiting our analyses to hospitals with homogeneous ventilation indexes across their ORs. Further, the need for aggregating the ventilation quality across ORs precluded an individual evaluation of each index component’s influence on the occurrence of SSIs. In addition, subgroup analyses in the hospital-level analyses resulted in small numbers, which limited our power to detect differences. In consequence, patient-level analyses with increased statistical power (but with the limitation that no direct link between patient and OR can be made) were performed in a second step, which largely confirmed the hospital-level findings. Finally, we cannot exclude the possibility that some hospitals reported only data on their best ORs. However, relying on an overly optimistic exposure is more likely to bias our results toward the null, and therefore would imply that the true association between ventilation index and SSI rates would be even stronger.

In conclusion, our results indicate that performing orthopedic and cardiac interventions in ORs with good OR ventilation properties (as assessed using a novel ventilation index proposed here) is associated with lower rates of superficial and deep incisional SSIs. In contrast, ventilation might play a minor role in other surgical procedures. The beneficial impact of OR ventilation quality on the subset of superficial and deep incisional infections needs confirmation in other cohorts. Further studies should prospectively include data on specific aspects of a given OR that allow examining the influence of each component of the ventilation index on SSI detected in patients operated on in this particular room. Finally, cost-benefit analyses taking our findings into account are needed to further delineate the role of OR ventilation on the occurrence of SSIs.

## Supplementary Material

SUPPLEMENTARY MATERIAL
