# Supplementary material for: Better Operating Room Ventilation as Determined by a Novel Ventilation Index is Associated With Lower Rates of Surgical Site Infections
Source: Ann Surg. 2022 Aug 10;276(5):e353–60. doi: 10.1097/SLA.0000000000005670 (PMC9534050; doi:10.1097/SLA.0000000000005670)
Supplement: SUPPLEMENTARY MATERIAL [file sla-276-e353-s001.docx]

**SUPPLEMENTARY MATERIAL**

**Figure S1** Questionnaire sent out to all participating hospitals (*next pages*)


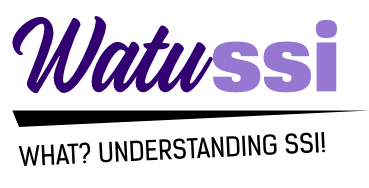


Watussi-Study «Operating room ventilation and SSI rates» – Survey Form

# *Please fill out one form per operating room*

# A) General information about the operating room (OR)

Hospital Name:

| OP-ID: |  | *Unique identifier for the OR* |
| --- | --- | --- |

| Types of procedures regularly performed in this OR (multiple answers possible): | | The OR has been used for this type of procedure since month/year (mm/yyyy): |
| --- | --- | --- |
| ☐ | Appendectomy |  |
| ☐ | Cholecystectomy |  |
| ☐ | Colon Surgery |  |
| ☐ | Rectal Surgery |  |
| ☐ | Gastric Bypass Surgery |  |
| ☐ | Hernia Repairs |  |
| ☐ | Cesarean Section |  |
| ☐ | Hysterectomy |  |
| ☐ | Laminectomy / Spondylodesis |  |
| ☐ | Cardiac Surgery |  |
| ☐ | Elective Hip Arthroplasty |  |
| ☐ | Elective Knee Arthroplasty |  |

| Surface area of the operating room (approx.): |  | m2 | Room height of the operating room (approx.): |  | m |
| --- | --- | --- | --- | --- | --- |

| OR with anesthetic room? | ☐ | yes | ☐ | no |
| --- | --- | --- | --- | --- |

| Fixation of the operating table: | ☐ | stationary | ☐ | movable |
| --- | --- | --- | --- | --- |

Number of doors of the OR:

# B) General information about the operating room ventilation system

Ventilation system in operation since month/year (mm/yyyy):

Frequency of testing air flow rate & overflow: ☐ every year ☐ every 2 years ☐ every 3 years ☐ every 4 years

| Is a protected area marked on the floor? | | ☐ | yes | ☐ | no |  |  |  |
| --- | --- | --- | --- | --- | --- | --- | --- | --- |
| 🡪 *If yes*, size of protected area: | Length 1: | |  |  | m | Length 2: |  | m |

| Filter class of the last supply air filter stage | ☐ | F9 | ☐ | H13 | ☐ | H14 | ☐ | Other: |
| --- | --- | --- | --- | --- | --- | --- | --- | --- |

Does the OR have positive pressure? ☐ yes ☐ no *Test: put a thin paper towel at the door gap: If it is*

*moving out = positive pressure*

Total supply air flow for the OR: m3/h Outside air flow in the supply air: m3/h

| Air exchange rate: |  | 1/h |
| --- | --- | --- |

| Location of exhaust air/overflow openings: | ☐ | unten | ☐ | oben |
| --- | --- | --- | --- | --- |


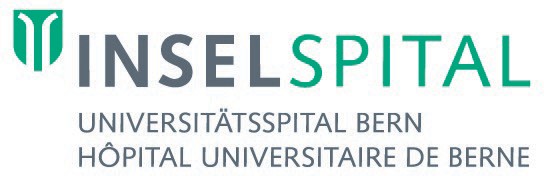

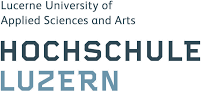

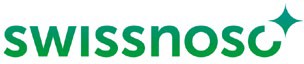

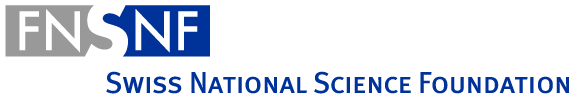


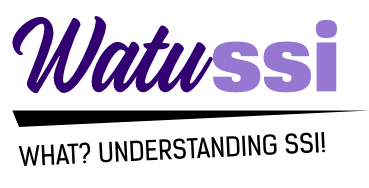


# C) Specific data for laminar air flow (SWKI-concept 1a)

*Feature: one large air unit above the OR-table*

Form and size of laminar air flow unit ☐ Rectangular 🡪 Length 1:

- Octacon 🡪 Diagonal:
- Circle 🡪 Diameter:

m Length 2: m m

m

Air guide at ceiling unit: *circumferential frame around the laminar air flow unit*

Type: ☐ none ☐ Guide with media supply bridge ☐ Guide without media suppley bridge

- Number of open sides ☐ none ☐ one ☐ two
- Height: m *Distance from the surgical ceiling to the lower edge of the guide*

OR Lights:

- Manufacturer:
- Type:
- Quantity:

Degree of protection achieved at last measurement: With OR lights in place

Without OR lights in place

Date of measurement: month/year (mm/yyyy):

*Information on the manufacturer and type can be read directly from the operating light.*

*The degree of protection measures the relative protection of the zone under the air flow unit against a particle load located outside of it.*

# D) Specific data for conventional (turbulent) air flow (SWKI-concept 1b)

*Feature: several smaller air diffusers instead of one large air flow unit*

| Supply air outlets: |  |  |  |  |  |  |
| --- | --- | --- | --- | --- | --- | --- |
| - Type: | ☐ | Round outlets | ☐ | Quadratic outlets | ☐ | Outlets in wall |
| - Quantity: |  | Ceiling: | Wall: | |  |  |

| Recovery time at last measurement: |  | Minutes | *The '1:100' recovery time measures the time it takes to reduce the level of particulates to one hundredth of the pollution peak produced by the introduction of a sample charge.* |
| --- | --- | --- | --- |
|  |  |  |  |
| Date of measurement: month/year (mm/yyyy): |  |  |  |

# E) 2 Photos of the Operating Room


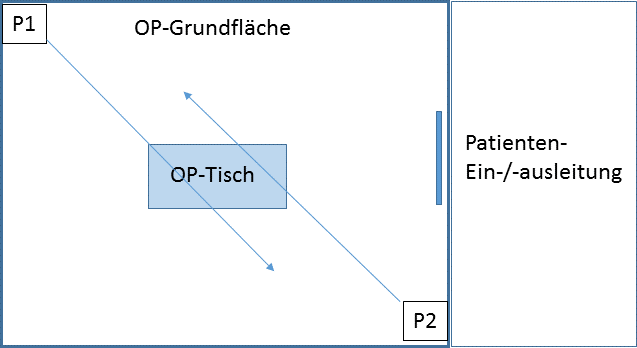
In addition to the information on this form, please take 2 pictures of the operating room ceiling so that the following objects are visible: Supply air diffuser/ diffusers, operating room lights, air flow guide, ceiling supply units. The photos should show the objects from opposite positions (P1+P2). Please make sure that no persons or patient data (e.g. on a monitor) are visible on the pictures. Please name the photos with the OR-ID, i.e. the code you used for the OR on the first page of the form. The images are used for data validation by the Lucerne University of Applied Sciences and Arts.

Please send the completed form and 2 photos by email no later than March 25, 2020.
The data will be treated confidentially. Thank you for your support!

| **Contacts** |  |
| --- | --- |
| - for organizational questions: | Judith Maag, [judith.maag@extern.insel.ch](mailto:judith.maag@extern.insel.ch) |
| - for technical questions: | Benoit Sicre, [benoit.sicre@hslu.ch](mailto:benoit.sicre@hslu.ch) |


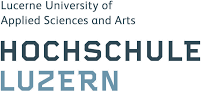

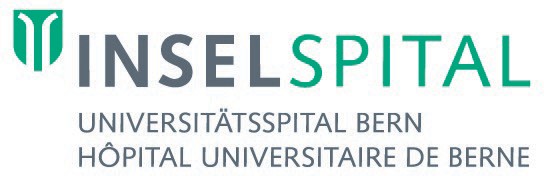
2


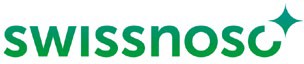

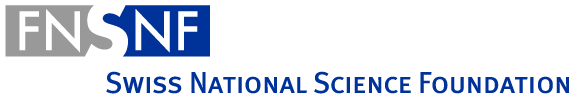


### **Figure S1** Ventilation indexes of all operating rooms from participating hospitals, stratified by type of surgery performed

## **
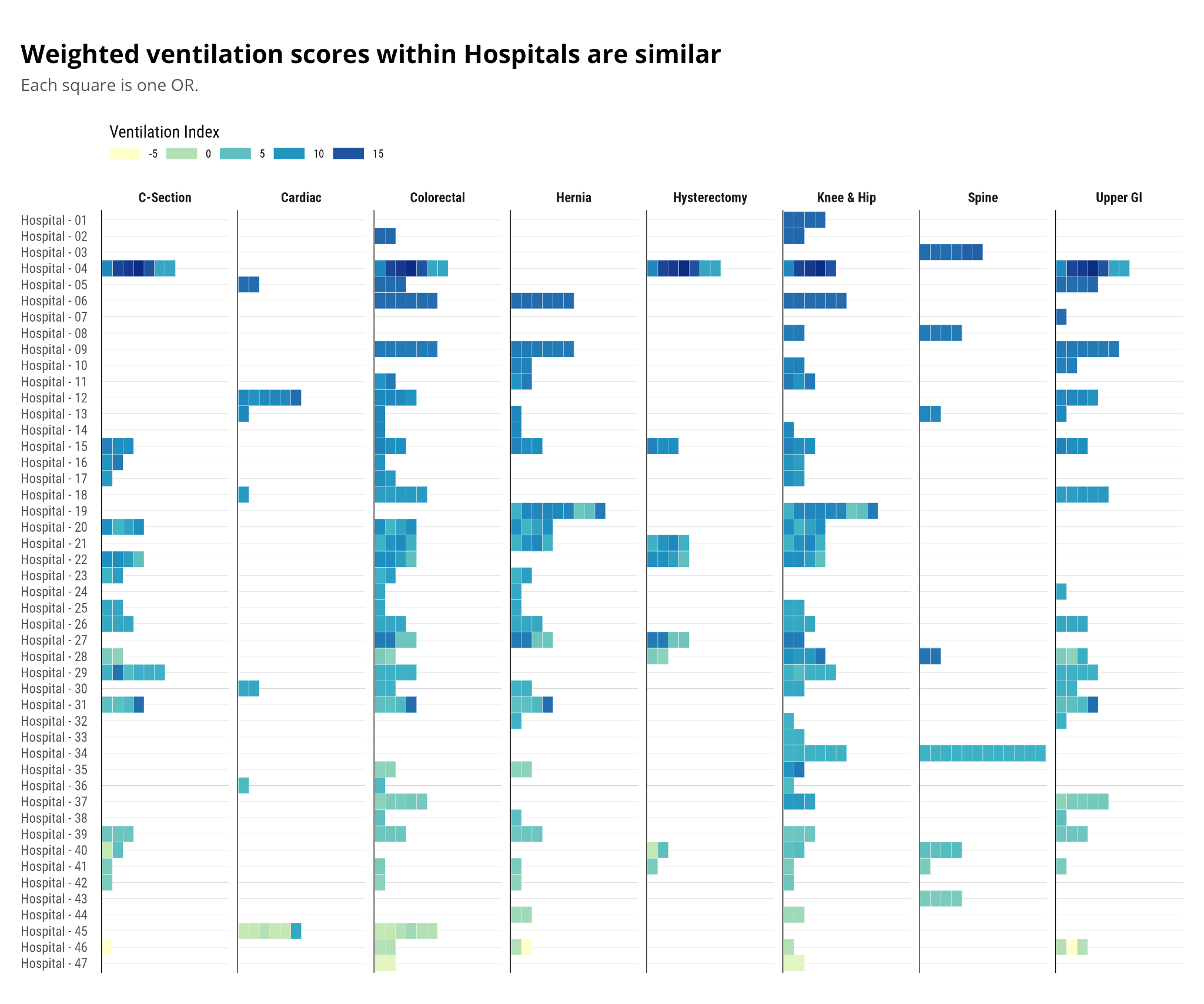
**

Each square indicates a distinct operating room (OR) within a hospital. Higher ventilations indexes reflect better laminar air flow quality. **GI** = gastrointestinal. **C-section** = cesarean section.

### **Table S1** Patient characteristics of the patient-level analysis

| **Variable** | **Overall (n = 163’740)** |
| --- | --- |
| Female sex (%) | 82’577 (50.4) |
| Median age, *years* (IQR) | 63.9 (49.7 to 73.5) |
| ASA physical status class (%) |  |
| 1 | 18’315 (11.2) |
| 2 | 79’389 (48.5) |
| 3 | 49’690 (30.3) |
| 4 | 15’114 ( 9.2) |
| 5 | 494 ( 0.3) |
| *missing* | *738 ( 0.5)* |
| Procedure (%) |  |
| Cesarean section | 15’963 ( 9.7) |
| Cardiac | 30’121 (18.4) |
| Colorectal | 22’751 (13.9) |
| Hernia repair | 17’097 (10.4) |
| Hysterectomy | 2914 ( 1.8) |
| Knee & hip arthroplasty | 54’088 (33.0) |
| Spine | 6119 ( 3.7) |
| Upper GI | 14’687 ( 9.0) |
| Surgery type (%) |  |
| clean | 105’500 (64.4) |
| clean-contaminated | 41’818 (25.5) |
| contaminated | 10’256 ( 6.3) |
| dirty | 6163 ( 3.8) |
| *missing* | *3 ( 0.0)* |
| Median duration of intervention, *min* (IQR) | 95 (64 to 162) |
| Elective intervention (%) | 138’532 (84.6) |
| No. of hospital beds of the facility (%) |  |
| less than 200 | 75’408 (46.1) |
| 200-499 | 37’771 (23.1) |
| 500 or more | 50’561 (30.9) |
| Type of hospital (%) |  |
| Private | 54’068 (33.0) |
| Public | 79’829 (48.8) |
| University | 29’843 (18.2) |
| **IQR** = interquartile range, **ASA** = American Society of Anesthesiologists,  **GI** = gastrointestinal, **min** = minutes, **No.** = number  *missing* is only shown if there are any missing observations for a given variable. | |

###

###

### **Table S2** Sensitivity analyses aggregating within hospital indexes using the minimum and maximum ventilation index

|  | **Change in infections per 100 interventions (95% CI)*** | |
| --- | --- | --- |
| **Surgery Type** | **Min. of indexes** | **Max. indexes** |
| Overall | -0.31 (-1.14 to 0.52) | -0.03 (0.43 to 0.37) |
| Cesarean Section | -0.02 (-0.56 to 0.53) | -0.10 (-0.51 to 0.30) |
| Cardiac | -0.82 (-1.74 to 0.10) | -1.27 (-3.38 to 0.84) |
| Colorectal | -0.41 (-2.07 to 1.25) | -0.25 (-1.71 to 1.22) |
| Hernia repairs | -0.02 (-0.35 to 0.31) | -0.01 (-0.31 to 0.29) |
| Hysterectomy | 0.10 (-1.40 to 1.61) | 0.41 (-0.60 to 1.42) |
| Knee & Hip arthroplasty | -0.34 (-0.63 to -0.04) | -0.39 (-0.64 to -0.14) |
| Spine | -1.16 (-2.58 to 0.26) | -1.11 (-2.51 to 0.29) |
| Upper GI | -0.32 (-1.00 to 0.36) | -0.19 (-0.91 to 0.54) |

*Changes in infection rates, per 5 units in the ventilation index.

**GI** = gastrointestinal, **CI** = confidence interval, **min** = minimum, **max** = maximum
